# Effect of Two Choreographed Fitness Group-Workouts on the Body Composition, Cardiovascular and Metabolic Health of Sedentary Female Workers

**DOI:** 10.3390/ijerph16244986

**Published:** 2019-12-07

**Authors:** Yaira Barranco-Ruiz, Robinson Ramírez-Vélez, Antonio Martínez-Amat, Emilio Villa-González

**Affiliations:** 1 Department of Physical and Sports Education, PROFITH “PROmoting FITness and Health through physical activity” Research Group, Sport and Health University Research Institute (iMUDS), Faculty of Education and Sport Sciences, University of Granada, 52071 Melilla, Spain; evilla@ugr.es; 2 Navarrabiomed-Universidad Pública de Navarra (UPNA)-Complejo Hospitalario de Navarra (CHN), Instituto de Investigación Sanitaria de Navarra (IdiSNA), 31008 Pamplona, Navarra, Spain; robin640@hotmail.com; 3 Department of Health Sciences, Faculty of Health Science, University of Jaen, 23071 Jaen, Spain; amamat@ujaen.es

**Keywords:** Physical activity, Cardiovascular Risk, Prevention, Women, Adults

## Abstract

Daily sedentary working hours contribute negatively to body composition, cardiovascular and metabolic health, especially in women, who are usually less active than men. The objective of this study was to analyze the effect of two trending choreographed fitness group-workouts on the body composition and cardiovascular and metabolic health of sedentary female workers. A total of 98 physically inactive and working women (38.9 ± 6.4 years of age) were randomly assigned to three study groups: Control group (CG) = 31, Zumba Fitness^®^ with three one-hour classes per week (ZF) = 39, and Zumba Fitness with 20 min of additional Bodyweight strength training (ZF + BW) = 28. Measurements included body composition, blood pressure, cardiovascular risk by the Framingham Heart Study tools (10 yr cardiovascular risk and vascular age) and a metabolic blood panel. Post-intervention, both choreographed fitness group-workouts reached a similar significant loss of fat mass (ZF = 2.805 ± 0.48, *p* < 0.0001; ZF + BW = 3.540 ± 0.04, *p* < 0.0001), an increase in muscle mass (ZF = 1.70 ± 0.581, *p* = 0.005; ZF + BW = 3.237 ± 0.657, *p* < 0.0001) and a decrease in SBP (ZF= 6.454 ± 1.70, *p* < 0.0001; ZF + BW = 4.12 ± 1.95, *p* = 0.039). Only the ZF group significantly improved the 10 yr cardiovascular risk (*p* = 0.032) and metabolic age (*p* = 0.0025) post-intervention. No significant improvement was observed in the metabolic panel for both choreographed fitness group-workouts. In conclusion, the ZF program generated improvements in cardiovascular and metabolic risk variables compared to ZF + BW or CG. Both choreographed fitness group-workouts contributed similarly to the improvement in systolic blood pressure, fat mass, muscle mass, and also engendered a great adherence to exercise.

## 1. Introduction

A sedentary lifestyle negatively affects cardiovascular and metabolic health and is related to risk of morbidity and mortality [1]. Studies estimate that 60% of people worldwide are insufficiently physically active to obtain health benefits [2]. This is due to their low participation in physical activity during free time, as well as a high prevalence of sedentary tasks at workplaces throughout the day. This also increases the amount of daily sedentary time and results in low expenditure of energy [3], contributing to obesity and cardiovascular diseases [4]. 

Physical inactivity is more prevalent in women than in men, both in high-income countries (35% of physically inactive women versus 26% of men) and in low-income countries (24 % of physically inactive women versus 12% of men) [5]. It is thus essential to promote novel strategies for increasing physical activity (PA) that guarantee adherence and self-efficacy, especially in women [6]. It has been observed that group PA interventions can arouse feelings of social connection, which contribute to improvements in motivation, adherence, and the psychological well-being of participants [7]. Additionally, the inclusion of music and choreography in PA programs can be valuable in improving their social, motivational, and psychological health dimensions [8]. In 2018, the Office for Disease Prevention and Health Promotion published the second edition of the physical activity guidelines for Americans, which describes a campaign to combat physical inactivity called “Move your way”. Among the alternatives suggested to promote PA, group classes including choreographies to the rhythm of the music have been encouraged [9]. Choreographed fitness group classes such as Zumba Fitness (ZF) are presented as a strategy to promote PA in sedentary adults, especially in women, who show greater adherence to group fitness classes particularly when music is incorporated [10,11]. ZF has been considered an effective program to achieve the benefits received from completing aerobic PA recommendations made by the World Health Organization (WHO) [12]. To date, there have been numerous investigations into the benefits of ZF-based exercise interventions, displaying improvements in various aspects such as cardiovascular and metabolic health, body composition, quality of life [6], and emotional health [13]. 

The American College of Sports Medicine (ACSM) annually publishes a list of the 20 fitness trends for next year, incorporating a ranking of individual and group-based fitness classes with the greatest impact. For 2019, fitness modalities like bodyweight strength training were in the top positions in recent years in both group and individual formats. Bodyweight training is a convenient alternative, due to a lack of required materials or equipment and ease of intensity control through the progression of exercises, safeguarding the health of individuals [14]. In addition, this modality could be choreographed, and contributes to muscle strengthening, which should be added to weekly aerobic PA, as recommended by the WHO. However, more research is required to understand the effects of choreographed fitness group classes like ZF or Bodyweight training in sedentary working women.

The aim of this study was to analyze the effect of these two choreographed fitness group-workouts on the body composition and cardiovascular and metabolic health of sedentary female workers. This information could be relevant, as it could support these types of programs as positive strategies for preventing the risks associated with a sedentary lifestyle in women.

## 2. Materials and Methods 

### 2.1. Design

This is an experimental study with randomized clinical trial design and a 3 × 2 statistical design. The study consists of two experimental groups (two exercise interventions—Zumba Fitness and Zumba Fitness + bodyweight training), and a control group (no exercise intervention), as well as two evaluation moments, one before the interventions (pre-test) and the other at the end of them (post-test). The study design is oriented to analyze whether Zumba Fitness intervention and/or Zumba Fitness with bodyweight intervention improves health-related body composition and cardiovascular risk indicators compared with an inactive group.

### 2.2. Participants and Recruitment 

**Figure 1:**
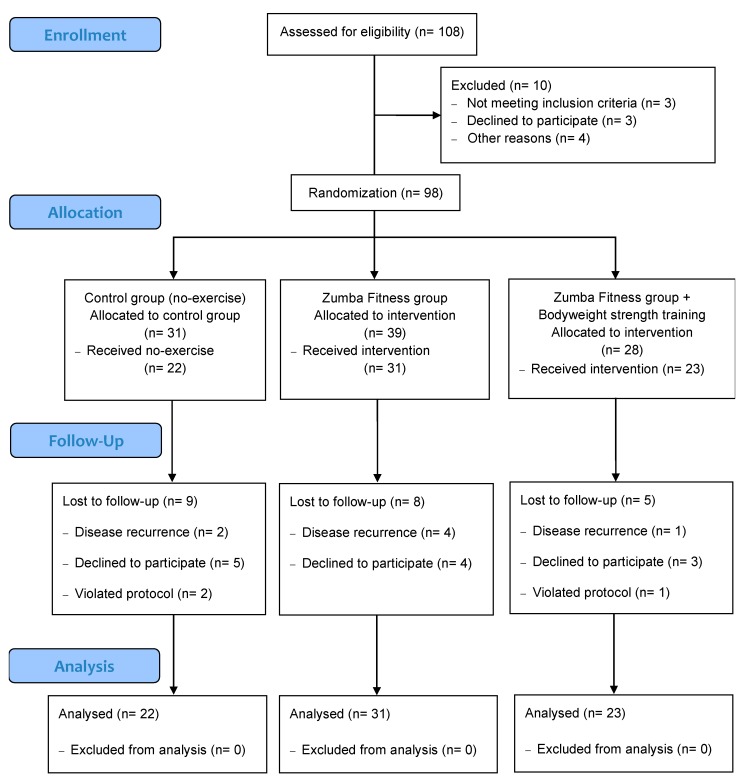
Flow diagram of the study participants.

This study is part of the "For a Healthy University" (Ref: 29-CI-2014-10-17-22) research project conducted at the National University of Chimborazo, Ecuador. The inclusion criteria were as follows: adult women (30 to 50 years old) who did not meet the minimum physical activity (PA) guideline according to WHO (i.e., perform less than 150 min of moderate–vigorous PA per week); participants must have no experience with group-based fitness programs such as Zumba Fitness^®^ classes or bodyweight training; participants must spend more than five hours per day sitting in their usual work tasks. The exclusion criteria were a diagnosis of cancer, skeletal muscle injuries, or serious cardiovascular diseases at the time of the study. The recruitment phase for the study began with an email to employees of all university campuses with brief information about the project and an invitation to attend initial informative meetings; a total of 948 potential participants were contacted. A total of 150 employees attended the two information sessions. Attendees of the sessions that demonstrated interest in participating filled out a registration form with personal contact information and data for initial eligibility screening. A total of 120 employees agreed to participate in the study. Of those 120 participants, 111 met the inclusion criteria of the study and were called for initial evaluation. A total of 108 participants attended evaluations to start the study; thirteen dropped out prior to starting the interventions. Ninety-eight participants were included in the intervention study (Figure 1).

After the initial evaluations, participants were randomly assigned to one of three groups: Control Group (GC), ZF, and Zumba Fitness + Bodyweight group (ZF + BW). After accounting for patient drop-outs, the groups were distributed with the following final counts: CG = 31, ZF = 39, and ZF + BW = 28 (Figure 1). The average participant age across all groups was 39 ± 6.4 years. Regarding study groups, the age average was 38.19 ± 5.6, 39.87 ± 7 and 39.21 ± 6.3, respectively. Randomization was blinded to the researchers; it was performed by an academic non-member of the research team. A numeric code was written on a ballot for each participant and situated in a central box next to three additional boxes, each corresponding to an intervention group. Someone unaffiliated with the research team randomly took a ballot from the central box and placed it in one of the three intervention boxes. Researchers did not know the numeric code assigned to the participant or the intervention assigned to each box. The ballot was always deposited in a left-to-right direction. 

### 2.3. Procedures

All participants performed 16 weeks of intervention outside working hours. The “For a Healthy University” project included two different exercise groups: the ZF and ZF + BW. All participants performed the ZF sessions at the same time and the ZF + BW group continued with an extra 20 min of muscular strength training. Both exercise interventions were carried out at 18:00, three days per week. The control group continued with their usual lifestyle. The three groups received two sessions of nutritional education throughout the project (Weeks 1 and 8) based on recommendations for healthy nutrition elaborated by a nutritionist.

### 2.4. Interventions

ZF intervention were led by a certified instructor and lasted one hour per session. Each class was divided into a warm-up of approximately 10 min with choreographed movements for articular mobility and dynamic stretching, followed by a 40 min section where the different Latin rhythms of merengue, salsa, reggaeton, and cumbia were combined. A final cool-down (5–10 min) of softer salsa or bachata rhythms, dynamic stretching, and breathing exercises concluded the session. The ZF + BW group carried out additional muscle strengthening with choreographed exercises involving a basic patter of movements through global movements. Participants trained with their own bodyweight, focusing on five muscle zones: lower limbs, chest, upper limbs, abdomen, and lower back. Muscle groups were always exercised in this order, using global strength exercises such as squats, lunges, chest and triceps push-ups, crunch-up, isometric abdomen exercises, and lower-back extensions. Participants performed a workout of approximately four minutes for each major muscle group, including variations in the speed of each movement. There was no rest time within the work of each muscle group and 30 s of rest was taken between changes in muscle group. This workout is similar to collective fitness exercise programs tested in previous studies, but without additional materials [15]. For both interventions, the same choreographies and exercise dynamics were used during the 16 weeks of intervention. In order to analyze the consistency of the intervention, the adherence and intensity of the intervention was measured. Adherence was measured by attendance to all intervention sessions. The intensity of the sessions was evaluated using the Borg scale (0–10) through the subjective perception of the participants’ effort [16] according to previous similar studies [17,18,19]. 

### 2.5. Outcome Measures

Body composition was carried out in the university medical center by qualified health professionals. Participants fasted for longer than 12 h and did not exercise for at least 48 h prior to beginning the initial evaluations. All participants underwent an anthropometric evaluation using the procedures described by the International Society for the Advancement of Kinanthropometry (ISAK), using the variables and methodology for the ISAK restricted profile [14] evaluating the following: skin folds, body perimeters, bone diameter, weight, and size. The thickness of the skin fold was recorded with a constant precision of 10 g/mm^2^ pressure using a Holtain skinfold gauge. (Holtain Ltd., Crymych, UK). The evaluation was carried out by a certified evaluator in ISAK level II, who was blinded to the participants’ group. Body mass index (BMI) was calculated as the weight (kg) divided by the squared height (m); BMI classification standards were adjusted to the provisions of the Consensus of the Spanish Society for Obesity Research (Spanish Society for the Study of Obesity) (BMI ≤ 25 = Normal-weight, BMI = 25–30 = Overweight, BMI = 30–35 = Obesity I, BMI > 35 = Obesity II) [20]. Waist–hip index (WHI) was calculated from the coefficient between the waist and hip perimeters expressed in centimeters, using the protocol reported by the World Health Organization [21]. Body fat mass and body muscle mass were calculated according to the guidelines of the Statement of the Spanish group of Kinanthropometry of Spanish Federation of Sports Medicine, using the equations that were valid for sedentary women [22]. Body fat mass was estimated through the Durnin & Womersley equations, where body density values (BD) were obtained, taking into account sex and the range of age [23]; subsequently, fat mass percentage was calculated using the Siri equation (%Fat mass = (495/BD) − 450). Muscle skeletal mass (Kg) was estimated by Lee equation [24]. 

Blood pressure was measured by trained nursing staff using a kit of arm sphygmomanometer and a stethoscope (Riester, Germany). The following blood pressure ranges were considered normal and without indication of cardiovascular risk [25]: Systolic blood pressure (SBP) ≤ 120 mmHg and diastolic blood pressure (DBP) ≤ 80 mmHg.

The 10-year cardiovascular risk was estimated from the Framingham risk score, which was calculated using the risk factors of age, sex, total cholesterol, HDL-cholesterol, smoking history, blood pressure, and diabetes mellitus. This algorithm predicts the risk of coronary heart disease at 10 years [26]. The optimal value of cardiovascular risk at 10 years was obtained according to the characteristics of the study sample. The metabolic age was extracted from the “The Framingham Heart Study” tools [26]. 

Qualified nursing staff drew 3 mL blood samples from the antecubital vein using vacutainers. The blood was centrifuged to separate the plasma at 3500 rpm for 15 min at 4 °C (Centrifugal Hettich, 12 EBA, Tuttlingen, Germany). The samples were deposited into containers for analysis by spectrophotometry (spectrophotomer Humalyzer 3500, Human diagnostic, Wiesbaden, Germany). The blood concentrations of glucose, triglycerides, total cholesterol, uric acid, and creatinine were evaluated and quantified in reference to healthy normative values (glucose = 70 to 110 mg/dL, triglyceride = 30 to 220 mg/dL, total cholesterol = 120 to 200 mg/dL, uric acid = 2 to 7 mg/dL, and creatinine = 0.5 to 0.9 mg/dL).

### 2.6. Statistical Analysis

Per protocol statistical analyses were performed by IBM SPSS Statistics 22.0 (IBM Corp, Armonk, NY, USA). The results were expressed as means and standard deviation (SD) and/or standard error of the mean for quantitative variables, and as percentage for qualitative variables. To evaluate the distribution of the variables, the Kolmogorov Smirnov test was performed. Qualitative variables comparisons were carried out using the chi squared and McNemar tests. Due to the design of the study (3 × 2), a mixed factorial ANOVA was carried out to assess the effect of the intervention on the dependent variables, the main effects of the study factors, and the possible interactions between the study factors. The mean change in each group was reported as the estimated margin of the mean with adjustment for baseline value as covariate using a linear-mixed effects model. In addition to repeated measures analysis of variance, Bonferroni–Holm corrections were performed (*p* = 0.00083, CI = 99.917%. Partial eta-squared (ηp^2^) study group × evaluation time interaction was calculated as the between-group sum of squares divided by the total sum of squares and interpreted as follows: ‘small’ effect (0.01); ‘small-to-medium’ effect (0.01–0.10); ‘medium-to-large’ (0.10–0.25). The within-group effect size of the exercise interventions was calculated using ‘Cohen’s d’ and interpreted as follows: small effect (0.10); small-to-medium (0.10–0.25); and medium-to-large effect (≥ 0.25).

## 3. Results

### 3.1. Characteristics of Participants 

The final analysis demonstrated a 22.4% drop-out rate, including 76 total participants who completed the pre- and post-intervention evaluation, distributed as follows: Control (*n* = 22), ZF (*n* = 31), ZF + BW (*n* = 23) (Figure 1). The attendance percentages were ZF = 77.7 ± 6.7 % and ZF + BW = 78.2 ± 9.1 %. The average perceived intensity during the exercise sessions was ZF = 7.2 ± 0.7 points Borg scale and ZF + BW = 7.8 ± 0.6 points Borg scale. 

### 3.2. Changes in Body Composition Variables

**Figure 2:**
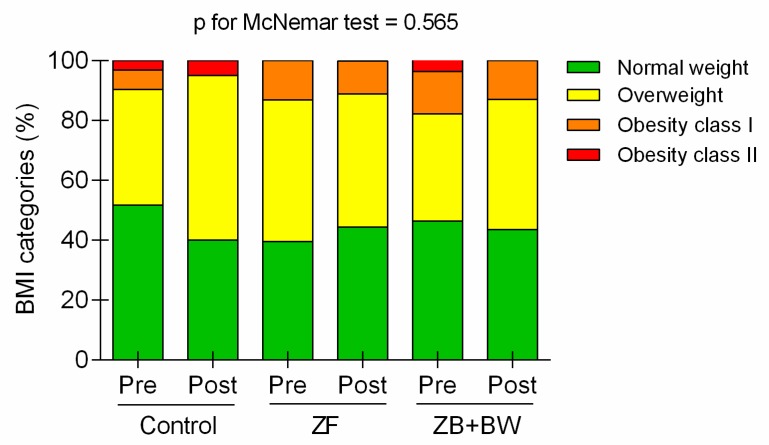
Body mass index changes by nutritional status. ZF= Zumba Fitness group; ZF + BW = Zumba Fitness + bodyweight group. Changes by McNemar test.

Body mass index changes by nutritional status are shown in Figure 2. The majority of the sample (the final participant count) presented as either normal weight (45.9%) or overweight (40.8%). Despite no statistically significant differences at baseline and post-intervention BMI assessments, a reduction in the prevalence of obesity I and II was observed at post-intervention for both exercise groups. Only the ZF group showed an increase in the number of participants within the normal-weight BMI category (4.9%). 

**Figure 3:**
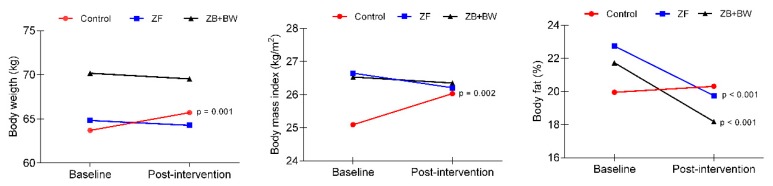
Significant interactions in anthropometric and body composition variables.

Significant interactions were observed for body weight (F _(1.73)_ = 7.911, *p* = 0.001, η^2^*p* = 0.209), BMI (F _(1.73)_ = 7.161, *p* = 0.002, η^2^*p* = 0.176) and fat mass (F _(1.73)_ = 11.063, *p* < 0.001, η^2^*p* = 0.269) and are presented in Figure 3. While the exercise intervention groups showed a reduction in these variables, the CG experienced an increase in them. Additionally, a significant main effect of time factor was observed for WHI (F _(1.73)_ = 3048.3, *p* < 0.001, η^2^*p* = 0.981) and muscle mass (F _(1.73)_ = 4.037, *p* = 0.049, η^2^*p* = 0.063). 

Changes related to baseline in anthropometric and body composition variables, according to the study group, are shown in Supplemental Table S1. Both exercise intervention groups experienced significant changes in WHI, fat mass (%) and muscle mass (Kg) after the period of intervention (16 weeks). Specifically, ZF and ZF + BW groups significantly increased in kg of muscle mass and significantly decreased WHI and the percentage of fat mass. Although the changes in fat mass (%) and muscle mass (Kg) were higher for the ZF + BW group, there were no significant differences between groups for these variables. However, the ZF + BW group showed a higher effect size on these variables, compared with the ZF group (Fat mass: ZF = 0.70 versus ZF + BW = 1.17; Muscle mass: ZF = 0.44 versus ZF + BW = 0.55). The CG also significantly decreased in WHI and increased muscle mass (Kg) after the intervention, but without differences between the rest of the groups. Additionally, the CG significantly increased in body weight and BMI at post-test, whereas the exercise groups reduced these variables, but without significant changes. 

### 3.3. Changes in Blood Pressure and Cardiovascular/Metabolic Health Outcomes

**Figure 4:**
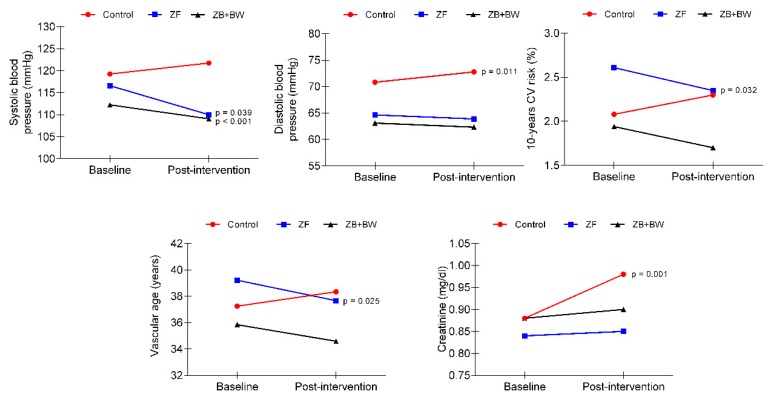
Significant interactions in blood pressure and cardiovascular/metabolic health variables.

Significant interactions were observed for SBP (F _(1.73)_ = 7.024, *p* = 0.002, η^2^*p* = 0.175), DBP (F_(1.73)_ = 4.580, *p* = 0.014, η^2^*p* = 0.252) 10-year cardiovascular risk (F _(1.73)_ = 5.925, *p* = 0.004, η^2^*p* = 0.154), vascular age (F _(1.73)_ = 4.056, *p* = 0.022, η^2^*p* = 0.114) and creatinine (F _(1.73)_ = 3.691, *p* = 0.031, η^2^*p* = 0.108), Figure 4. The ZF group experienced a significant decrease in cardiovascular health variables (10-year cardiovascular risk and vascular age), whereas the CG and ZF + BW groups did not show any changes. Likewise, the ZF group significantly decreased SBP, whereas the CG significantly increased it, and the ZF + BW group did not show a significant change. Finally, the CG significantly increased DBP and creatinine, whereas both exercise intervention groups did not show significant changes in these variables. Additionally, a significant effect of the time factor was observed in SBP (F_(1.73)_ = 5.275, *p* = 0.025, η^2^*p* = 0.074), 10-year cardiovascular risk (F_(1.73)_ = 4.609, *p* = 0.036, η^2^*p* = 0.066), glucose (F_(1.73)_ = 27.263, *p* < 0.001, η^2^*p* = 0.309) and creatinine (F_(1.73)_ = 13.090, p = 0.001, η^2^*p* = 0.177). Changes related to baseline blood pressure and cardiovascular/metabolic health variables, according to the study group, are shown in Supplemental Table S2.

## 4. Discussion

The aim of this study was to analyze the effect of two choreographed fitness group-workouts on body composition and cardiovascular and metabolic health in sedentary female workers. The main findings were that both exercise interventions generated a similar improvement in body composition and blood pressure values. However, only the ZF intervention, without an extra 20 min of bodyweight training, improved cardiovascular health variables, such as the percentage of 10-year cardiovascular risk and vascular age. The control group experienced several detriments, such as an increase in body weight, BMI, DBP, glucose and creatinine blood concentrations.

Concerning the health-related anthropometric and body composition variables that were investigated in this study, there was a high prevalence of participants at baseline who were in the BMI overweight category. However, after the intervention programs, the ZF group demonstrated a ~2% reduction in BMI risk prevalence. In quantitative terms, both exercise intervention groups experienced significant and similar decreases in the percentage of body fat mass (ZF = 2.805 ± 0.48, *p* < 0.0001, effect size = 0.70; ZF + BW = 3.540 ± 0.04, *p* < 0.0001, effect size = 1.17). These values are similar to previous intervention studies based on ZF in overweight Italian women [27]. In that study, the analysis of body composition changes by bioimpedance showed that body fat mass decreased by ~6.3%, with a notable concurrent increase in muscle mass. In our study, muscle mass significantly increased in both exercise intervention groups and, to a lesser extent, in the control group. The ZF + BW group significantly increased kg of muscle mass post-intervention (3.237 ± 0.657 kg), and the ZF group also had a significant post-intervention increase of 1.70 ± 0.581 kg. 

Other studies based on ZF intervention have demonstrated significant differences in both BMI and WHI, although each study utilized a different duration of intervention. Specifically, the following statistically significant reductions in BMI were observed using different intervention timelines: 3.7% after 12 weeks [27]; 1.1% after 16 weeks [28]; and 2.5% [29] and 1.8% [17] after eight weeks. Reductions in body perimeter were also found after 12 weeks of intervention [27], specifically in the circumferences of the waist (4.5%), hip (5%), arm (7.9%), and thorax (4%). Comparatively, Krishnan et al. [28] reported a decrease in waist (3.5%) and hip (2.3%) circumference after a 16-week ZF intervention. However, in the current study, no significant differences were found with respect to BMI in the exercise groups, since only control group significantly increased BMI, by 0.89 ± 0.27 kg/m^2^. In this sense, changes in fat mass and muscle mass compartments could explain the lack of change in post-intervention BMI. While BMI can be as, or more, clinically important than total adiposity measures as a predictor of cardiovascular disease mortality [30], people with a high muscle/low fat body mass have a lower risk of CVD and total mortality [31]. For that reason, we consider the improvements obtained in muscle and fat mass in this study to be beneficial for the cardiovascular and metabolic health of the participants.

Regarding blood pressure, and cardiovascular and metabolic health variables, both exercise intervention groups presented similar significant decreases in SBP (ZF = 6.454 ± 1.70, *p* < 0.001, effect size = 0.48; ZF + BW = 4.12 ± 1.95, *p* = 0.039, effect size = 0.41). [32]. Most of the studies with a Zumba Fitness intervention that analyzed the blood pressure as a cardiovascular health variable did not report any post-intervention changes [28,33,34], even with very extensive interventions (40 weeks) [33]. However, as in our study, two similar studies in adult women [11,27] found a significant decrease in systolic blood pressure after 12 weeks of ZF intervention program, with reductions of 13.63 and 6.7 mmHg, respectively. 

As a relevant result, only the ZF group showed significant improvements post-intervention in 10-year cardiovascular risk and estimated vascular age. To our knowledge, there are no studies in the literature on the effect of Zumba fitness exercise interventions on predictive cardiovascular risk variables through The Framingham Heart Study tools. However, studies that have included predominantly endurance exercise interventions, like ZF, have reported improvements in cardiovascular risk prediction variables [32]. 

According to the metabolic blood panel, no significant post-intervention changes were found in blood glucose, cholesterol, triglyceride, creatinine, and uric acid concentrations. Similarly, several studies based on ZF interventions in adult women did not observe significant improvements in these variables [27,28,34]. Only the Araneta et al. [11] observed a significant decrease in triglyceride concentration compared to pre-intervention values. However, it should be noted that these study participants were diagnosed with metabolic syndrome, whereas our sample contained apparently healthy women (with no established diseases such as diabetes or hypertension). It is important to highlight that Ecuadorian people usually have harmful nutrition habits, which could affect the results regarding the lack of change or detriment in metabolic blood profile [35,36], and maybe the two nutritional education sessions in our study were not enough to produce changes in nutrition habits. Another important note about our results is that participants in the ZF + BW group reported a significant increase in uric acid concentration values, without exceeding the healthy ranges. There are several causes that can influence the increase in post-exercise uric acid, but the best known is the influence of the intensity of the exercise [37,38]; at a greater intensity, there is greater degradation of nucleotides and a greater increase in uric acid, or urate in the blood. However, in our study, the intensity of the sessions was controlled by the subjective perception scale of Borg [16], reporting in this group an average of 7.8 ± 0.6, that corresponds to a moderate to vigorous intensity and not strenuous exercise. An alternative cause could be that some of the participants were in the menstruation cycle, which is related to an increase of 0.5 mg/dL. Unfortunately, the menstruation cycle was not controlled as a variable in our study. However, the increases in no case exceed the healthy range of uric acid concentration. In relation to the control group, it should be noted that they experienced significant increases in triglyceride and creatinine variables, two parameters closely and negatively linked to metabolic health.

Regarding the study’s practical applications, people who attend a gym or fitness center commonly think that, by performing an additional training of a few minutes (20–30), of bodyweight muscle strengthening exercises after one hour of ZF, they can achieve great improvements in body composition. However, it seems that additional bodyweight exercises do not contribute to losses of fat or increases in muscle beyond what is gained through ZF-like participation. So, it is possible that increases in the duration, intensity and/or density in the strength training, as in a concurrent training model, are required to achieve changes in body composition [39]. In terms of cardiovascular health, performing one hour of ZF could improve blood pressure compared with an additional 20 min of BW training. However, results indicated that neither of the group-based fitness programs generated changes in metabolic blood variables. Additionally, both fitness programs registered a similar attendance rate of ~80%, which means that both programs could generate increased adherence in this population and be used to improve body composition and prevent cardiovascular and metabolic risk in sedentary adult women. Although these results could be explained with different hypotheses, a fact highlighted and presented in the current study was that the ZF intervention was guided by a graduate in physical exercise sciences, also a certified instructor in Zumba Fitness modality (ZIN), which is an important fact that should be taken into consideration in future exercise interventions.

Finally, as a limitation of the study, the selection process was via convenience, although a randomized intervention assignment was done. Some variables are estimations of cardiovascular and metabolic risk; however, they are widely supported in the related literature. In terms of intensity control, we could have used a more objective measure, like pulsometer, however, we have used the Borg Scale (0–10) as a valid method, according to previous, similar studies mentioned in the methods section. As a strength, this is a study that analyzes two recent worldwide trends in fitness activities, which have been little-studied in association with the prevention of metabolic and cardiovascular risks. Additionally, intervention adherence was high, with approximately 80% session attendance; the participant drop-out rate was less than 25%. This is important, considering that a rate greater than 25% is considered a “fatal flaw” when evaluating public health-related intervention [40]. 

## 5. Conclusions

A 16 week-Zumba Fitness intervention, with one hour per session/three times per week, induced improvements in several cardiovascular health variables, such as the percentage of 10-year cardiovascular risk and vascular age, compared with the rest of the study groups (ZF + BW and control). Doing a ZF session with an extra 20 min of Bodyweight training generates similar improvements in SBP, fat mass and muscle mass as performing the ZF session only; additionally, both choreographed fitness group-workouts presented a high adherence to exercise. Conversely, the control group, with only two nutritional educational sessions and no exercise intervention, showed significant detriments in body weight, BMI, fat mass, DBP, and glucose/creatinine levels. New physical exercise strategies, such as choreographed fitness group-workouts trends, should be analyzed and used to combat the cardiovascular and metabolic health risks due to physical inactivity in women.

## Supplementary Materials

The following are available online at https://www.mdpi.com/1660-4601/16/24/4986/s1, Tables S1 and S2.
