# Supplementary material for: Effect of Two Choreographed Fitness Group-Workouts on the Body Composition, Cardiovascular and Metabolic Health of Sedentary Female Workers
Source: Int J Environ Res Public Health. 2019 Dec 7;16(24):4986. doi: 10.3390/ijerph16244986 (PMC6950037; doi:10.3390/ijerph16244986)
Supplement: Supplementary file 1 [file ijerph-16-04986-s001.docx]

**Effect of Two Choreographed Fitness Group-Workouts on the Body Composition, Cardiovascular and Metabolic Health of Sedentary Female Workers**

Yaira Barranco-Ruiz ^1*^, Robinson Ramírez-Vélez ^2^, Antonio Martínez-Amat ^3^, and Emilio Villa-González ^1^

**Table 1.** Changes in anthropometric and body composition variables according to study groups.

| **Anthropometric and body composition variables** | **Control group (n=22)** | | | | |  | **ZF (n=31)** | | | | |  | **ZF + BW (n=23)** | | | | | |
| --- | --- | --- | --- | --- | --- | --- | --- | --- | --- | --- | --- | --- | --- | --- | --- | --- | --- | --- |
|  | Marginal mean diff (SE diff) | | | P value | Effect size |  | Marginal mean diff (SD diff) | | | P value | Effect size |  | Marginal mean diff (SE diff) | | | P value | Effect size | |
| Body Weight (Kg) | -1.967 | ± | 0.55 | **0.001** | 0.25 |  | 0.59 | ± | 0.44 | 0.188 | 0.05 |  | 0.55 | ± | 0.06 | 0.270 | 0.05 |  |
| Height (m) | 0.00 | ± | 0.01 | 0.515 | 0.02 |  | 0.00 | ± | 0.00 | 0.624 | 0.05 |  | 0.00 | ± | 0.00 | 0.416 | 0.04 |  |
| BMI (Kg/m2) | -0.89 | ± | 0.27 | **0.002** | 0.25 |  | 0.42 | ± | 0.23 | 0.073 | 0.10 |  | 0.17 | ± | 0.04 | 0.509 | 0.05 |  |
| WHI | .090 | ± | 0.02 | **0.000** | 0.46 |  | .123 | ± | 0.02 | **0.000** | 0.09 |  | .101 | ± | 0.01 | **0.000** | 0.01 |  |
| Fat mass (%) | -0.07 | ± | 0.60 | 0.908 | 0.13 |  | 2.805 | ± | 0.48 | **0.000** | 0.70 |  | 3.540 | ± | 0.04 | **0.000** | 1.17 |  |
| Muscle mass (Kg) | -1.47 | ± | 0.71 | **0.041** | 0.43 |  | -1.70 | ± | 0.581 | **0.005** | 0.44 |  | -3.237 | ± | 0.657 | **0.000** | 0.55 |  |
| BMI = Body Mass Index. WHI = Waist-Hip Index. ZF = Zumba Fitness intervention group. ZF + BW = Zumba Fitness + 20 minutes of Body weight training.  The mean change in each group was reported as the estimated margin of the mean and standard error differences (SE) using baseline value as covariate. CI = 99.917% through a mixed factorial ANOVA. Statistical significance Bonferroni adjustment p = 0.00083. **P value in bold** = significant differences regarding baseline values. | | | | | | | | | | | | | | | | | | |

| **Cardiovascular and metabolic health variables** | **Control group (n=22)** | | | | | **ZF (n=31)** | | | | | | **ZF + BW (n=23)** | | | | |
| --- | --- | --- | --- | --- | --- | --- | --- | --- | --- | --- | --- | --- | --- | --- | --- | --- |
|  | Marginal mean diff (SE diff) | | | P value | Effect size | Marginal mean diff (SE diff) | | | P value | Effect size | Marginal mean diff  (SE diff) | | | | P value | Effect size |
| ***Blood Pressure*** |  |  |  |  |  |  |  |  |  |  |  | |  |  |  |  |
| SBP (mmHg) | -3.32 | ± | 2.04 | 0.108 | 0.21 | 6.454 | ± | 1.70 | **0.000** | 0.48 | 4.12 | | ± | 1.95 | **0.039** | 0.41 |
| DBP (mmHg) | -4.65 | ± | 1.78 | **0.011** | 0.22 | 1.39 | ± | 1.38 | 0.317 | 0.09 | 2.25 | | ± | 1.59 | 0.163 | 0.14 |
| ***Cardiovascular health*** |  |  |  |  |  |  |  |  |  |  |  | |  |  |  |  |
| 10-years cardiovascular risk (%) | -0.20 | ± | 0.11 | 0.072 | 0.22 | 0.21 | ± | 0.09 | **0.032** | 0.09 | 0.28 | | ± | 0.10 | 0.072 | 0.14 |
| Vascular age (years) | -1.08 | ± | 0.73 | 0.147 | 0.14 | 1.46 | ± | 0.64 | **0.025** | 0.14 | 1.36 | | ± | 0.74 | 0.071 | 0.26 |
| ***Metabolic blood panel*** |  |  |  |  |  |  |  |  |  |  |  | |  |  |  |  |
| Glucose (mg/dl) | -4.44 | ± | 2.59 | **0.000** | 0.10 | -3.85 | ± | 1.91 | 0.048 | 0.66 | -1.55 | | ± | 2.20 | 0.483 | 0.31 |
| Triglycerides (mg/dl) | 10.09 | ± | 49.77 | 0.840 | 0.20 | -11.38 | ± | 38.83 | 0.770 | 0.12 | -107.32 | | ± | 45.82 | **0.022** | 0.45 |
| Cholesterol (mg/dl) | 5.53 | ± | 10.80 | 0.611 | 0.18 | -8.66 | ± | 8.34 | 0.303 | 0.25 | -15.05 | | ± | 9.89 | 0.133 | 0.45 |
| Uric Acid (mg/dl) | -0.43 | ± | 0.25 | 0.097 | 0.37 | 0.16 | ± | 0.20 | 0.417 | 0.03 | -0.50 | | ± | 0.24 | **0.037** | 0.26 |
| Creatinine (mg/dl) | -.106 | ± | 0.03 | **0.001** | 0.76 | -0.01 | ± | 0.02 | 0.811 | 0.12 | -0.02 | | ± | 0.03 | 0.425 | 0.08 |
| SBP = systolic blood pressure, DBP = diastolic blood pressure. ZF = Zumba Fitness intervention group. ZF + BW = Zumba Fitness + 20 minutes of Body weight training.  The mean change in each group was reported as the estimated margin of the mean and standard error differences (SE) using baseline value as covariate. CI = 99.917% through a mixed factorial ANOVA. Statistical significance Bonferroni adjustment p = 0.00083. **P value in bold** = significant differences regarding baseline values | | | | | | | | | | | | | | | | |

**Table 2.** Changes in blood pressure and cardiovascular/metabolic health variables.
